# Pancreatic alpha cells in diabetic rats express active GLP-1 receptor: Endosomal co-localization of GLP-1/GLP-1R complex functioning through intra-islet paracrine mechanism

**DOI:** 10.1038/s41598-018-21751-w

**Published:** 2018-02-27

**Authors:** Koji Nakashima, Hideaki Kaneto, Masashi Shimoda, Tomohiko Kimura, Kohei Kaku

**Affiliations:** Department of Diabetes, Endocrinology and Metabolism, Kawasaki Medical School, 577 Matsushima, Kurashiki, 701-0192 Japan

## Abstract

Glucagon-like peptide-1 (GLP-1) stimulates insulin secretion from pancreatic beta cells and suppresses glucagon secretion from alpha cells. It remains controversial, however, whether GLP-1 receptor (GLP-1R) is expressed in mature alpha cells. In this study, unlike previous studies using non-diabetic animals, we demonstrated using diabetic model rats and confocal laser scanning microscopy that the GLP-1/GLP-1R complex was located in the endosome of diabetic islets. In addition, we showed that GLP-1 and GLP-1R co-localized with various endosomal markers and adenylate cyclase in the alpha cells of diabetic rats. Diabetic rats had endosomal signaling pathway but normal rats had classical signaling pathway for activated GLP-1R. Furthermore, we performed pancreatic perfusion to assess the functional activity of GLP-1R when stimulated by exendin-4 (EX4). In a pancreas perfusion study, EX4 significantly stimulated glucagon secretion in diabetic rats but not normal rats. However, such glucagon secretion was immediately suppressed, probably due to concomitantly secreted insulin. The GLP-1/GLP-1R complex appears to function through an intra-islet paracrine mechanism in diabetic conditions which could explain, at least in part, the mechanism of paradoxical hyperglucagonaemia in type 2 diabetes.

## Introduction

Glucagon-like peptide-1 receptor (GLP-1R) is a pharmacological target of incretin therapy for type 2 diabetes. Incretin-related agents, such as glucagon-like peptide-1 receptor (GLP-1R) agonist and dipeptidyl peptidase-4 (DPP-4) inhibitors, are widely used to treat diabetic patients^1,2^.

In non-diabetic conditions, pancreatic alpha cells mainly produce glucagon, which is a counter-regulatory hormone to insulin, so that homeostasis of glucose metabolism is maintained. It is known that alpha cells also synthesize small amounts of GLP-1^3–5^. We have recently reported that GLP-1 is secreted in a pulsatile fashion that is independent of GLP-1 secretion from the intestine^6^, although it remains unknown how GLP-1 secreted from alpha cells functions.

In contrast, under diabetic conditions (e.g., beta cell injury), alpha cells can proliferate and upregulate the expression level of GLP-1 as a growth and survival factor for injured beta cells and transdifferentiate to insulin-secreting beta cells^7,8^. It is also known that alpha cells increase the production of glucagon under diabetic conditions, leading to aggravation of diabetes^9–14^. Indeed, paradoxical glucagon secretion is observed in hyperglycaemic type 2 diabetes^15–17^. It has been reported that GLP-1 and GLP-1R agonist secrete insulin from beta cells, which suppresses glucagon secretion from alpha cells via an intra-islet paracrine mechanism^18^. It has been disputed whether GLP-1R is expressed in the alpha cells of adult islets^19–27^, although several researchers reported that GLP-1 exerts some effects through GLP-1R receptor in alpha cells^28,29^, suggesting the presence of GLP-1R in alpha cells. It is also important to manage undesirable paradoxical glucagon elevation under diabetic conditions using paracrine suppression of glucagon by insulin.

G protein-coupled receptors (GPCRs) such as GLP-1R are difficult to detect immunologically using standard immunocytochemistry and western blotting^30–33^. In addition, in the above controversial discussion about whether GLP-1R is present and works in pancreatic alpha cells, all researchers used normal animals. However, there could be an advantage in performing the experiments using diabetic model animals based on recent discoveries about the alpha cell transdifferentiation to beta cells found in diabetes^7,8^ and the presence of GLP-1R in foetal pancreatic alpha cells^28^. Recently endosomal-signaling transduction of family B GPCRs has been reported in addition to classical signaling pathway^34–37^. We hypothesized that GLP-1R expression would be induced in alpha cells under diabetic conditions and that the GLP-1/GLP-1R complex would be detectable in endosomes, rather than in the cell membrane. In this study, we attempted to detect the GLP-1/GLP-1R complex using a devised endosomal co-localization study and measured insulin, glucagon and GLP-1 through a pancreas perfusion study via the stimulation of exendin-4 (EX4).

## Results

### Concept of endosomal co-localization and pancreas perfusion study

The aim of this study was to examine whether GLP-1R is present in alpha cells under diabetic conditions. First, we presented endosomal co-localization in pancreatic islets of the GLP-1/GLP-1R complex, which is thought to be functional GLP-1R, and confirmed that this co-localization study was useful to detect the GLP-1/GLP-1R complex. Since we used no detergent or antigen retrieval reagent in this endosomal co-localization study to avoid destroying the structure of endosomes, in this system, it is very difficult to detect the expression of GLP-1R, which should be present on beta cell membranes. Next, using this co-localization study, we demonstrated that alpha cells in diabetic rats but not non-diabetic rats have the GLP-1/GLP-1R complex. Finally, to further examine the significance of GLP-1R in alpha cells, we performed a pancreatic perfusion study. In this study, we demonstrated that the GLP-1R agonist EX4 induced glucagon secretion from alpha cells in diabetic rats. These data strongly suggest that alpha cells have GLP-1R under diabetic conditions.

### The GLP-1/GLP-1R complex is co-localized with various endosome markers in pancreatic islets

**Figure 1:**
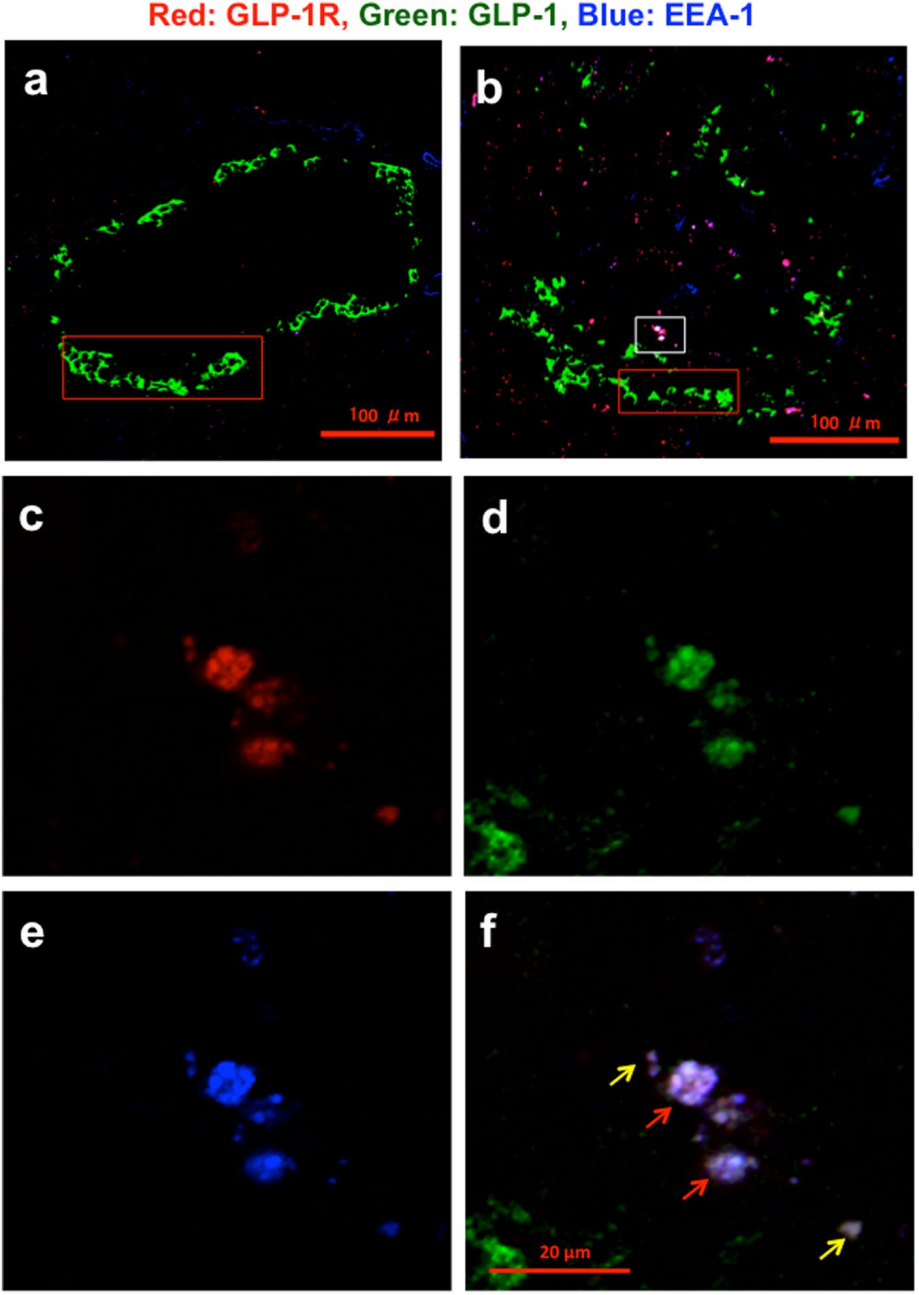
Co-localization of red GLP-1R, green GLP-1 and blue EEA-1 in islets of non-diabetic and diabetic rats. Confocal laser microscopy (CLM) images in a (**a**) 25- week-old non-diabetic Wistar rat (BW 489 g, FBS 119 mg/dl) and (**b**) 25-week-old diabetic GK rat (BW 370 g, FBS 210 mg/dl) were taken at 20× magnification. The white rectangular area containing large endosomes in Fig. 1b was taken at 64× magnification and was split into red GLP-1R (**c**), green GLP-1 (**d**) and blue EEA-1 (**e**). Figure 1f is a merged image of Fig. 1c–e. The yellow and red arrows indicate small and large endosomes, respectively. The red rectangular areas in Fig. 1a and b are shown again in Fig. 4a–d. Since we used no detergent or antigen retrieval reagent in this endosomal co-localization study to avoid destroying the structure of endosomes, in this system, it is very difficult to detect the expression of GLP-1R, which should be present in beta cell membrane. The epitope of GLP-1R in endosomes is preserved and recognized even without any detergent or antigen retrieval reagent. Therefore, the method in this study specifically detects GLP-1R in endosomes.

To examine the localization of GLP-1R in islets, we performed immunocytochemistry using F344/Jcl and Wistar rats treated or untreated with streptozotocin (STZ) and diabetic Goto-Kakizaki (GK) rats. The islet morphology of 5-week-old GK rats had a mantle arrangement of alpha cells similar to that of the non-diabetic Wistar rats, but the mantles were ruptured after 10 weeks of age (Fig. 1b). When GK rats had diabetes after 15 weeks of age, the ring-shaped alpha cell arrangement was destroyed with diffusely scattered alpha cells (Supple. Figure 1a). This image was also different from that of STZ-treated rats, in which islets had hyperplastic alpha cells and loss of beta cells (Supple. Figure 1b). In contrast, the non-diabetic Wistar rats maintained the islet morphology with the mantle arrangement of alpha cells, containing beta cells inside alpha cells until 25 weeks of age (Fig. 1a).

**Figure 2:**
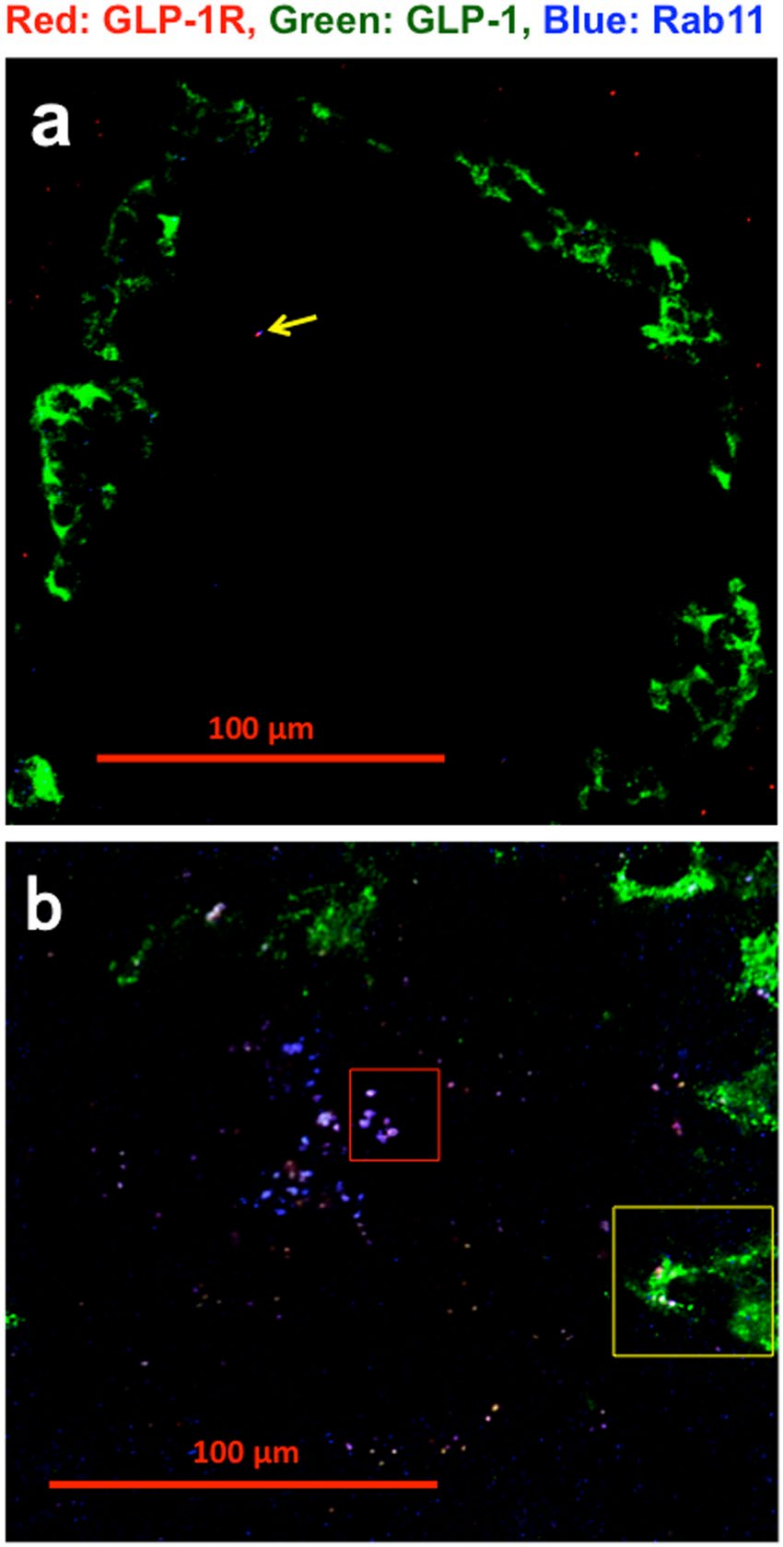
Co-localization of red GLP-1R, green GLP-1 and blue Rab11 in islets of non-diabetic and diabetic rats. CLM images of a (**a**) 25-week-old non-diabetic Wistar rat (BW 489 g, FBS 119 mg/dl) and (**b**) 25-week-old diabetic GK rat (BW 419 g, FBS 230 mg/dl) taken at 20× magnification. The yellow arrow in Fig. 2a indicates a small red dot in non-alpha cells. The red rectangular area in Fig. 2b is split and shown again in Supple. Figure 4. The yellow rectangular area in Fig. 2b is shown again in Fig. 4e and f.

We used these rats in the following immunohistochemical studies. First, we confirmed that GLP-1 was co-stained with glucagon using merged and split images of glucagon and GLP-1 (Supple. Figures 1 and 7), showing that alpha cells actually express GLP-1 as reported previously^3–5^. Because alpha cells express GLP-1^3–5^, the green color region indicates alpha cell area. Figure 1a and b show the confocal laser microscopy (CLM) images of islets in non-diabetic Wistar rats and diabetic GK rats, respectively. A few red dots (GLP-1R) were observed in the islets in non-diabetic rats (2 small dots have no GLP-1) (Fig. 1a). In diabetic GK rats, we found the co-localization of GLP-1, GLP-1R and early endosome auto-antigen 1 (EEA-1)^38^, which increased in number and size (3 large endosomes have GLP-1, 1 large endosome has no GLP-1, 4 small endosomes have GLP-1, but 7 small endosomes have no GLP-1) (Fig. 1b and Supple. Figure 2). In addition, we evaluated how often GLP-1 was positive in the endosomes using original images and Photoshop cs3. As the results, Co-localization of GLP-1 and GLP-1R was observed in 59% of early endosomes, but GLP-1 was not detected in 41% of early endosomes. We assume that the hybrid organelle endolysosome was formed in GLP-1-negative endosome as a part of endosome maturation, which led to the digestion of GLP-1.

**Figure 3:**
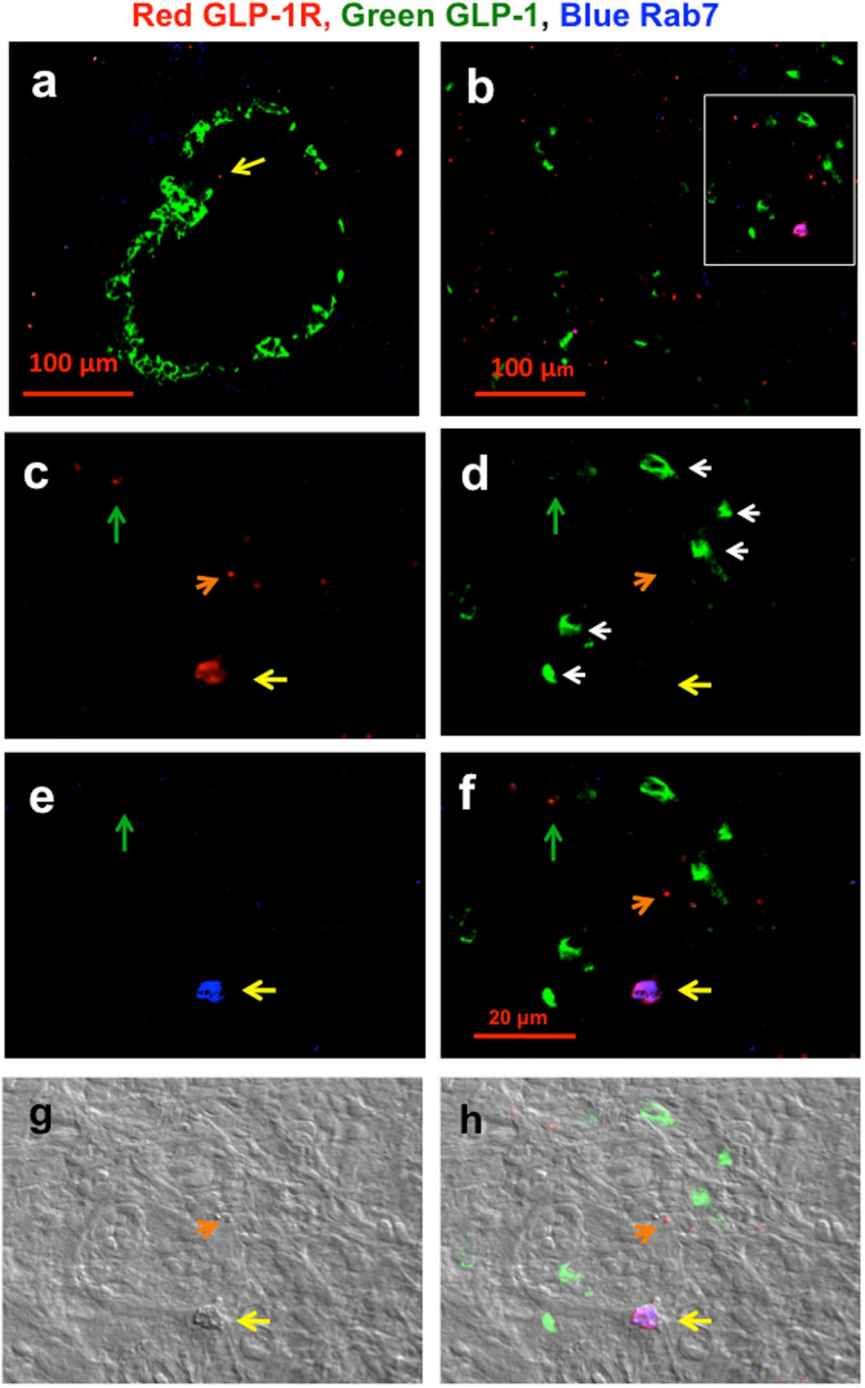
Co-localization of red GLP-1R, green GLP-1 and blue Rab7 in islets of non-diabetic and diabetic rats. CLM images in a (**a**) 25-week-old non-diabetic Wistar rat (BW 489 g, FBS 119 mg/dl) and (**b**) 25-week-old diabetic GK rat (BW 450 g, FBS 249 mg/dl) were taken at 20× magnification. The yellow arrow in Fig. 2a indicates a small red dot in non-alpha cells. The CLM image in the white rectangular area in Fig. 3b was taken at 63× magnification and split into red GLP-1R (**c**), green GLP-1 (**d**) and blue Rab7 (**e**). Figure 3f is a merged image of Fig. 3c–e. Figure 3g is a differential interference contrast (DIC) microscopy image that was taken simultaneously. Figure 3h is a merged image of Fig. 3f and g. The yellow and orange arrows in Fig. 3c–h indicate large and small endosomes, respectively. The green arrows in Fig. 3c–h indicate one of the small endosomes. The white arrows in Fig. 3d indicate cytoplasmic GLP-1-positive alpha cells.

The white rectangle area in Fig. 1b was taken by a 63× objective lens and was split into (c) red GLP-1R, (d) green GLP-1, and (c) blue EEA-1. Figure 1f shows a merged image of the three colors shown in Fig. 1c–e, indicating co-localization of GLP-1 and GLP-1R in early endosomes. It seemed that large early endosomes (red arrow in Fig. 1f) were produced by the fusion of small endosomes (yellow arrow). In addition, it is known that as the first step of internalization, the GLP-1/GLP-1R complex is recruited by beta arrestin. Indeed, as shown in Supple. Figure 3, white dots indicate a complex of GLP-1, GLP-1R and beta arrestin 2^39,40^.

Endosome with GLP-1/GLP-1R complex was scarcely detectable in the beta cell area of normal rats (Fig. 1a), compared to diabetic rats (Fig. 1b). In the normal islets it does not perform endocytosis to form endosomal GLP-1/GLP-1R complex (endosomal signaling pathway). GLP-1 signal transduction might be completed with GLP-1R on the cell membrane^40^ (classical signaling pathway)^34–37^.

**Figure 4:**
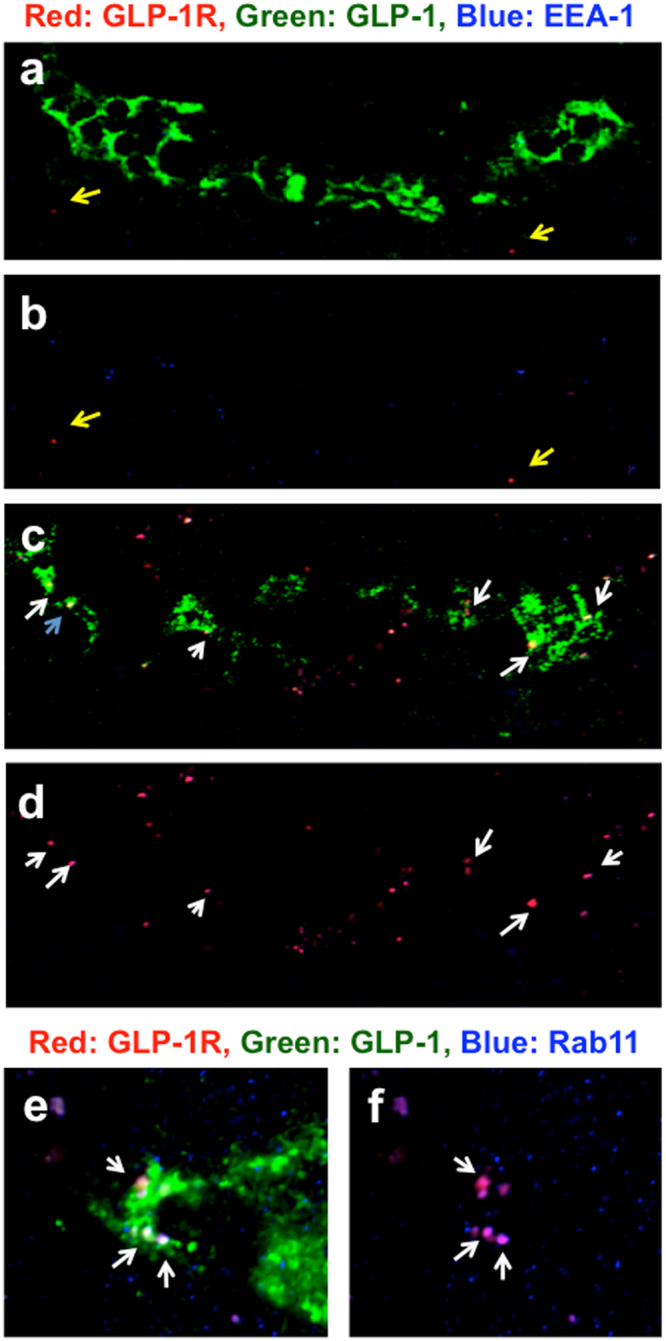
Endosomal co-localization of the GLP-1/GLP-1R complex in alpha cells in non-diabetic and diabetic rats. Figure 4a was taken from the red rectangular area in Figs 1a and 4b is a merged image of the red GLP-1R and blue EEA-1. Figure 4c was taken from the red rectangular area in Figs 1b and 4d is a merged image of the red GLP-1R and blue EEA-1. Figure 4e was taken from the yellow rectangular area in Fig. 2b with red GLP-1R, green GLP-1 and blue Rab11, and Fig. 4f is a merged image of the red GLP-1R and blue Rab11 in Fig. 4e. The yellow arrows indicate red GLP-1 R, which is not located in alpha cells. The white arrows in Fig. 4c and e indicate the co-localization of GLP-1R and endosome markers EEA-1 and Rab11, respectively, in alpha cells.

Next, we examined whether GLP-1R is recycled similarly to other GPCRs. Figure 2 shows CLM images of islets in 25-week-old non-diabetic Wistar rats and 25-week-old GK rats. Figure 2a shows a few endosomes (1 small endosome has GLP-1), but Fig. 2b shows many large and small endosomes in islets (3 large endosomes and 35 small endosomes have GLP-1, percentage of GLP-1-positive endosomes, 100%). The red rectangle area was photographed by a 63× objective lens and split to red GLP-1R, green GLP-1 and blue Rab11 in Supple. Figure 4. The blue Rab11^41,42^ is a marker of recycling endosomes. The intra-endosomal structures appeared to be different from those of early endosomes, suggesting the recovery of GLP-1R to the cell membrane.

We next examined late endosomes that fuse to lysosomes to digest GLP-1R and other proteins into amino acids and to synthesize new protein. We used the late endosome marker Rab7^43,44^. Figure 3a and b show representative CLM islet images of 25-week-old non-diabetic rats and 25-week-old diabetic GK rats, respectively. There were a few endosomes containing GLP-1R in islets in Fig. 3a (2 small endosomes have no GLP-1), but many large and small endosomes were observed in Fig. 3b (1 large endosome and 12 small endosomes have no GLP-1, and 1 small endosome has GLP-1 (green arrow) in Fig. 3c–f, (percentage of GLP-1-positive endosomes, 6%). In Fig. 3c–h, large and small endosomes (yellow and red arrows, respectively) were observed in the CLM images with (c) red GLP-1R, (d) green GLP-1 and (e) blue Rab7^43,44^, a late endosome marker. Although cytosolic GLP-1 was clearly stained in alpha cells (white arrow in Fig. 3d), GLP-1 was not detectable even in the large endosomes (yellow arrow in Fig. 3d). In addition, the differential interference contrast (DIC) microscope image (Fig. 3g) showed a three-dimensional structure of large and small endosomes that coincided with that in the CLM images (Fig. 3h).

### Alpha cells have the GLP-1/GLP-1R complex under diabetic conditions

To examine whether the GLP-1/GLP-1R complex is present in alpha cells, red rectangles of alpha cell area in Fig. 1a and b were further evaluated in Fig. 4a and c. In the magnified alpha cell area in the islet of a non-diabetic Wistar rat (Fig. 4a), white or magenta dots were not observed in alpha cells, but white dots were observed in the islet of a diabetic GK rat (Fig. 4c), indicating the co-localization of red GLP-1R, green GLP-1 and blue EEA-1 in alpha cells under diabetic conditions (Fig. 4c). In addition, as shown in Fig. 4d, magenta dots indicate the co-staining of red GLP-1R and blue Rab11. Figure 4e and f, which were taken from the yellow rectangle in Fig. 2b, show that the recycling endosomes contain the GLP-1/GLP-1R complex in alpha cells.

**Figure 5:**
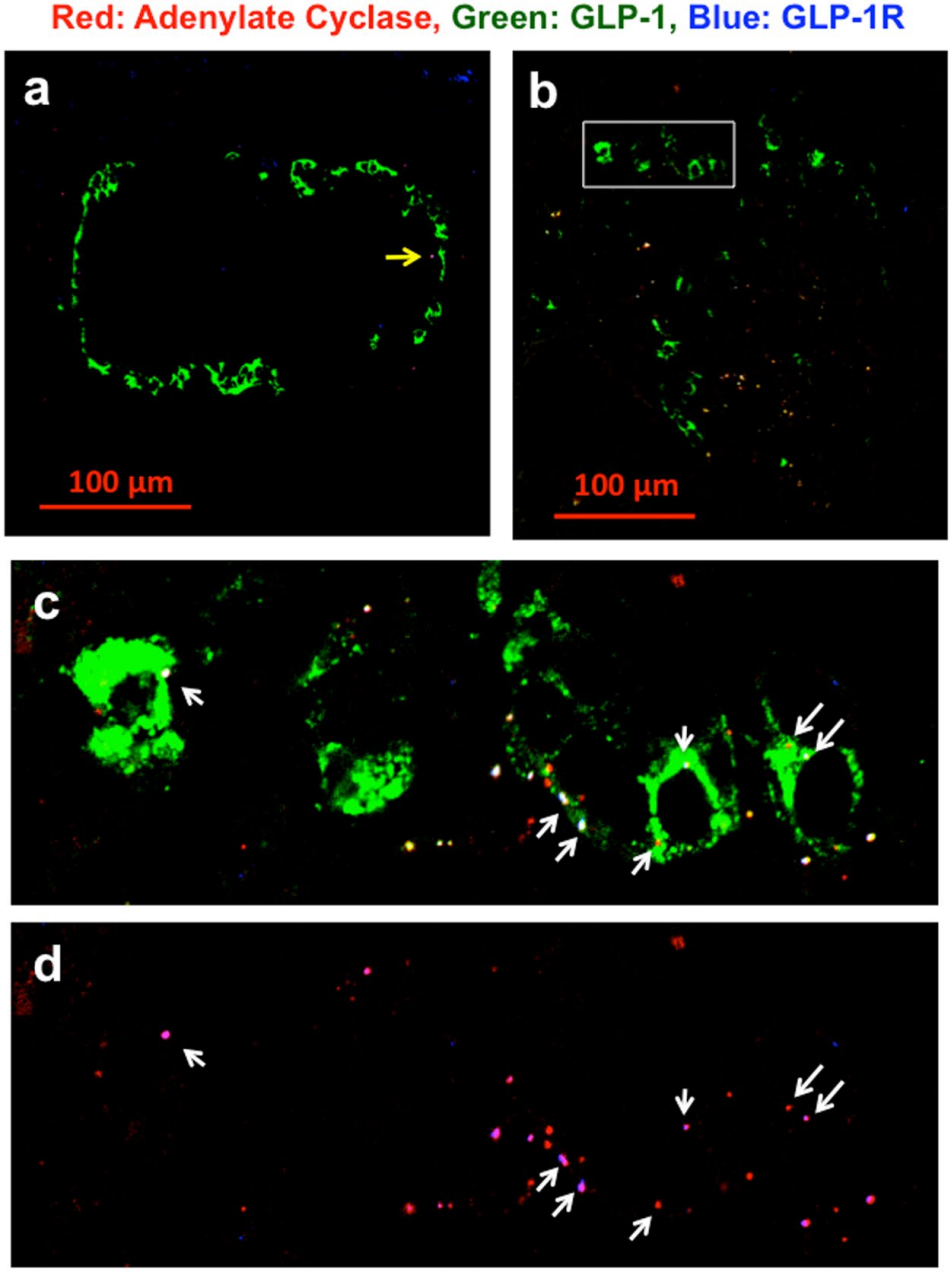
Co-localization of red adenylate cyclase (AC), green GLP-1 and blue GLP-1R. CLM images in a (**a**) 25-week-old non-diabetic Wistar rat (BW 487 g, FBS 119 mg/dl) and (**b**) 25-week-old GK rat (BW 370, FBS 125 mg/dl). The yellow arrows in Fig. 5a indicate a red dot in the non-alpha cell area. The white rectangular area in Fig. 5b was taken at 63× magnification and shown as Fig. 5c. Figure 5d is a merged image of AC and GLP-1R. The white arrows indicate the merging of AC and GLP-1R in alpha cells.

**Figure 6:**
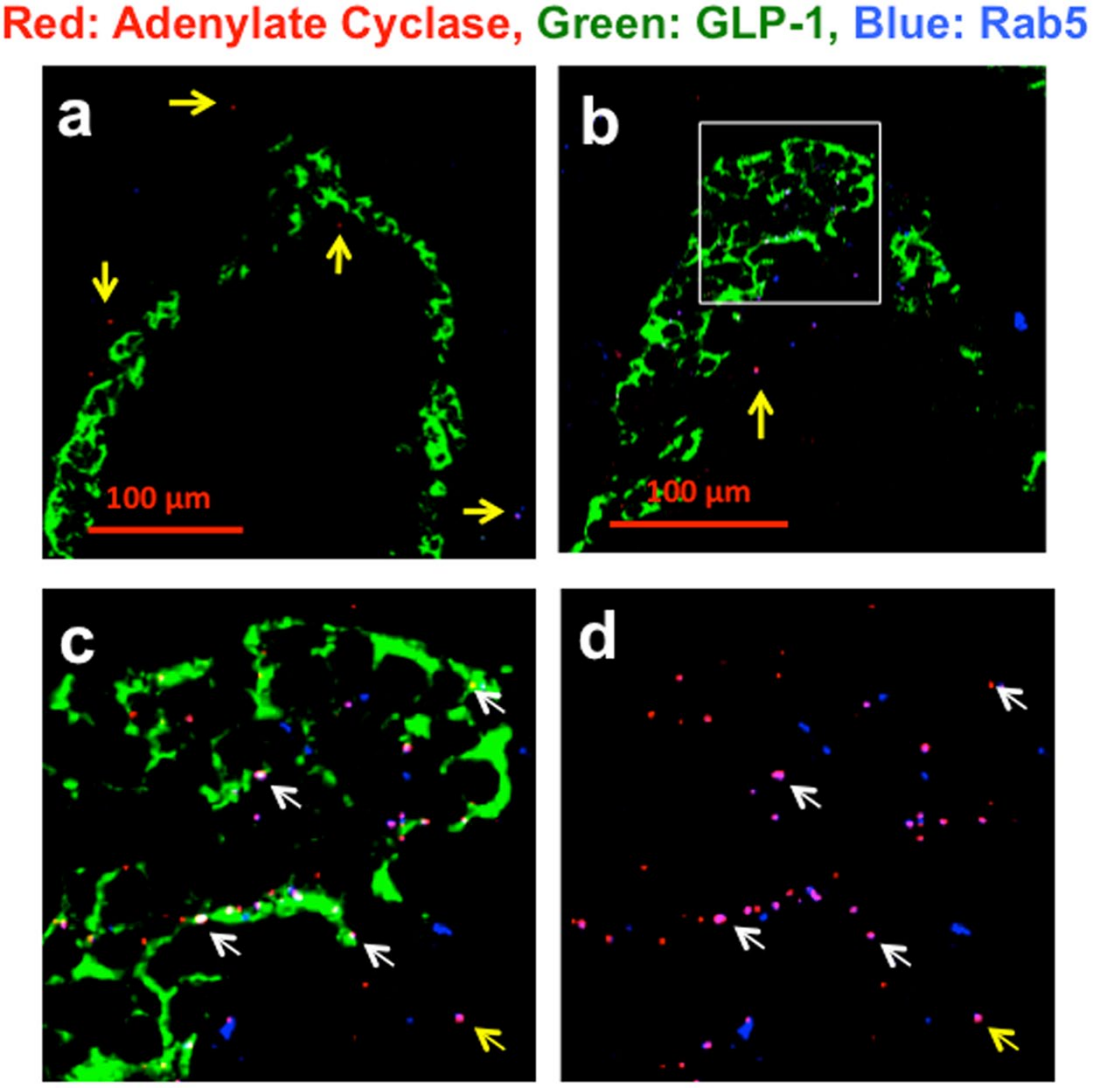
CML image of red AC, green GLP-1 and blue Rab5 in a (**a**) 10- week-old non-diabetic Wistar rat (BW 318 g, FBS 108 mg/dl) and (**b**) 10-week-old diabetic Wistar rat treated with STZ (BW 334, FBS 318 mg/dl). Figure 6c is a magnified image taken from the white rectangular area in Fig. 6b. Figure 6d is a merged image of the red AC and blue Rab5 in Fig. 6c. The yellow arrows indicate red AC, which is not located in alpha cells. The white arrows indicate the merging of AC and Rab7 in alpha cells.

In addition, adenylate cyclase (AC)^45^ was co-localized with GLP-1R and GLP-1 (Fig. 5). The white rectangle area in Fig. 5b was taken by a 63× objective lens and was presented in Fig. 5c. Merged image of red adenylate cyclase and blue GLP-1R was presented in Fig. 5d. The white dots indicated by white arrows demonstrated the co-localization of red AC, green GLP-1 and blue GLP-1R (Fig. 5c). Figure 6 shows the co-localization of AC, GLP-1 and Rab5, early endosome marker^46^ in STZ-induced diabetic Wistar rat. The images indicate that AC was incorporated in the early endosomes (Fig. 6a,b). The white rectangle of Fig. 6b was further examined in Fig. 6c,d. The white dots indicated by the white arrows demonstrate the co-localization of red AC, green GLP-1 and blue Rab5 (Fig. 6c). The magenta dots indicated by the white arrows in Fig. 6d are a merged image of red AC and blue Rab5. These data suggest the presence of an endosome containing AC, GLP-1 and GLP-1R that presumably functions as a signaling endosome.

### GLP-1R agonist facilitates glucagon secretion from alpha cells via GLP-1R

**Figure 7:**
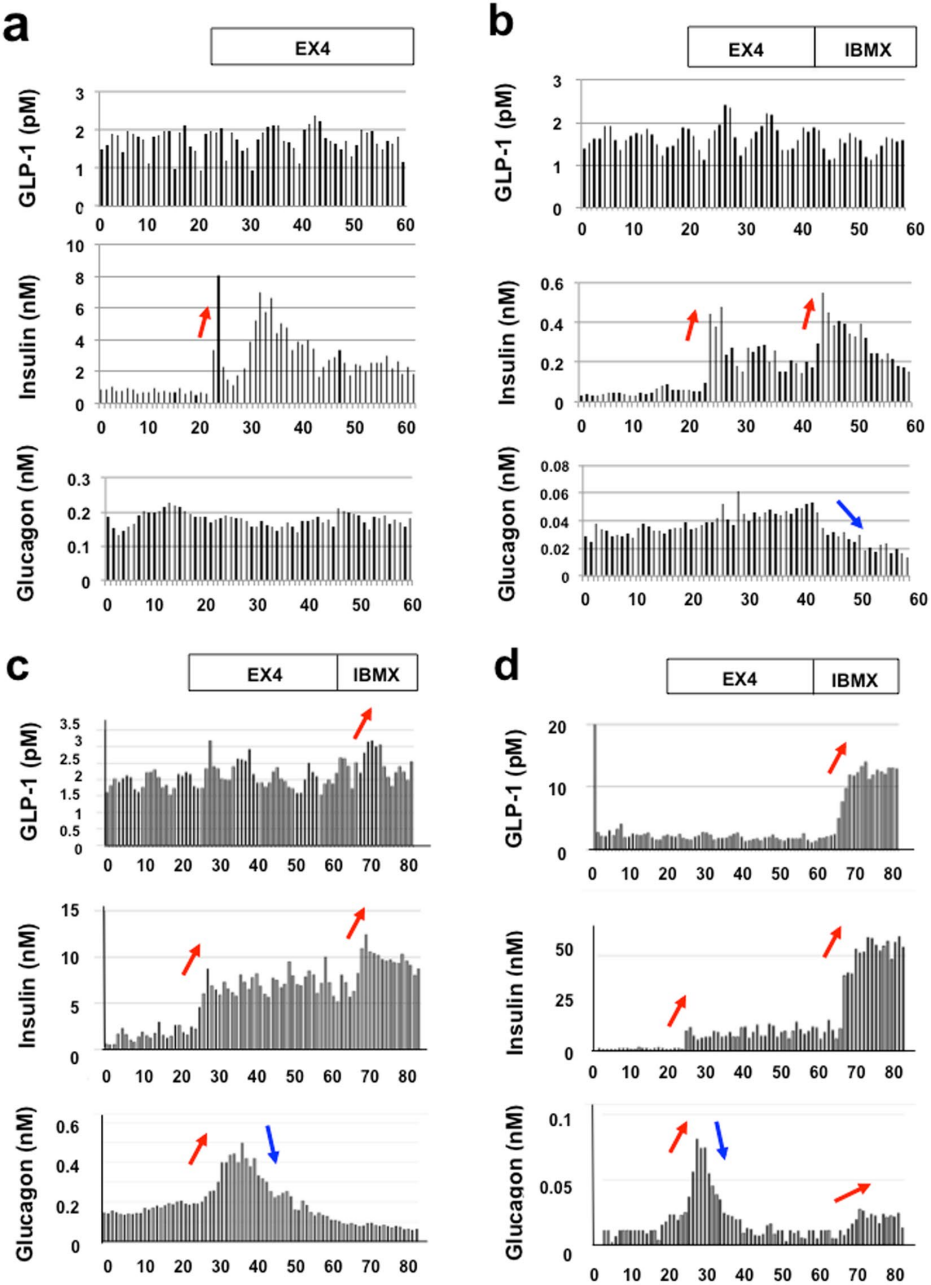
Isolated pancreatic perfusion profile of diabetic rats stimulated by EX4 and IBMX. (**a**) 11-week-old F344/Jcl rat (BW 276 g, FBS 110 mg/dl). 1–20 min, 5.5 mM glucose; 21–60 min, 5,5 mM glucose and 10 nM Exendin-4. (**b**) 12-week-old F344/Jcl rat treated with STZ (BW 194 g, FBS 291 mg/dl). 1–20 min, 5.5 mM glucose; 21–41 min, 5,5 mM glucose and 10 nM Exendin-4; 41–60 min, 5,5 mM glucose and 2.0 mM IBMX. (**c**) 10-week-old Wistar rat treated with STZ (BW 302 g, BS 472 mg/dl). 1–20 min, 5.5 mM glucose; 21–60 min, 5.5 mM glucose and 10 nM EX4; 61–80 min, 5.5 mM glucose and 2 mM IBMX. (**d**) 25-week-old GK rat (BW 370 g, BS 220 mg/dl). 1–20 min, 5.5 mM glucose; 21–60 min, 5.5 mM glucose and 10 nM EX4; 61–80 min, 5.5 mM glucose and 2 mM IBMX.

To further examine the significance of GLP-1R in alpha cells, we performed a pancreatic perfusion study using normal F344/Jcl (Fig. 7a), STZ-induced diabetic F344/Jcl (Fig. 7b) and STZ-induced diabetic Wister rats (Fig. 7c) and diabetic GK rats (Fig. 7d).

In normal control (Fig. 7a), EX4 stimulated insulin secretion but it did not secrete glucagon, suggesting that GLP-1R is present in beta cells but not in alpha cells. In order to see insulin paracrine action to suppress glucagon secretion, we used the cAMP-elevating agent 3-isobutyl-1-methylxanthine (IBMX, a phosphodiesterase inhibitor) after EX4 stimulation, because diabetic model rats might have less insulin secretion. As shown in Supple. Figure 13, IBMX stimulation led to the secretion of not only insulin but also GLP-1 and glucagon in diabetic rats. In normal control rats, however, there was no significant GLP-1 secretion in response to IBMX (Supple. Figure 12)^6^, suggesting that there is a marked difference between diabetic and non-diabetic alpha cells. As shown in Fig. 7b, EX4 stimulated insulin secretion in diabetic F344/Jcl rats. But glucagon level gradually elevated till IBMX stimulation induced additional insulin secretion and reciprocal suppression of glucagon. In Fig. 7b, insulin secreted by EX4 might not be sufficient to suppress the glucagon secretion. However, additional IBMX treatment induced enough insulin secretion to cause suppression of the glucagon secretion. EX4 markedly stimulated glucagon secretion (Fig. 7c,d), which strongly supported the idea that GLP-1R functions in glucagon-producing alpha cells under diabetic conditions. However, such marked glucagon secretion was subsequently sharply suppressed (Fig. 7c,d), presumably due to the insulin secretion from beta cells after EX4 treatment as reported previously^18^. In addition, the paracrine suppression of glucagon secretion by insulin was partially relieved by IBMX (Fig. 7d).

**Figure 8:**
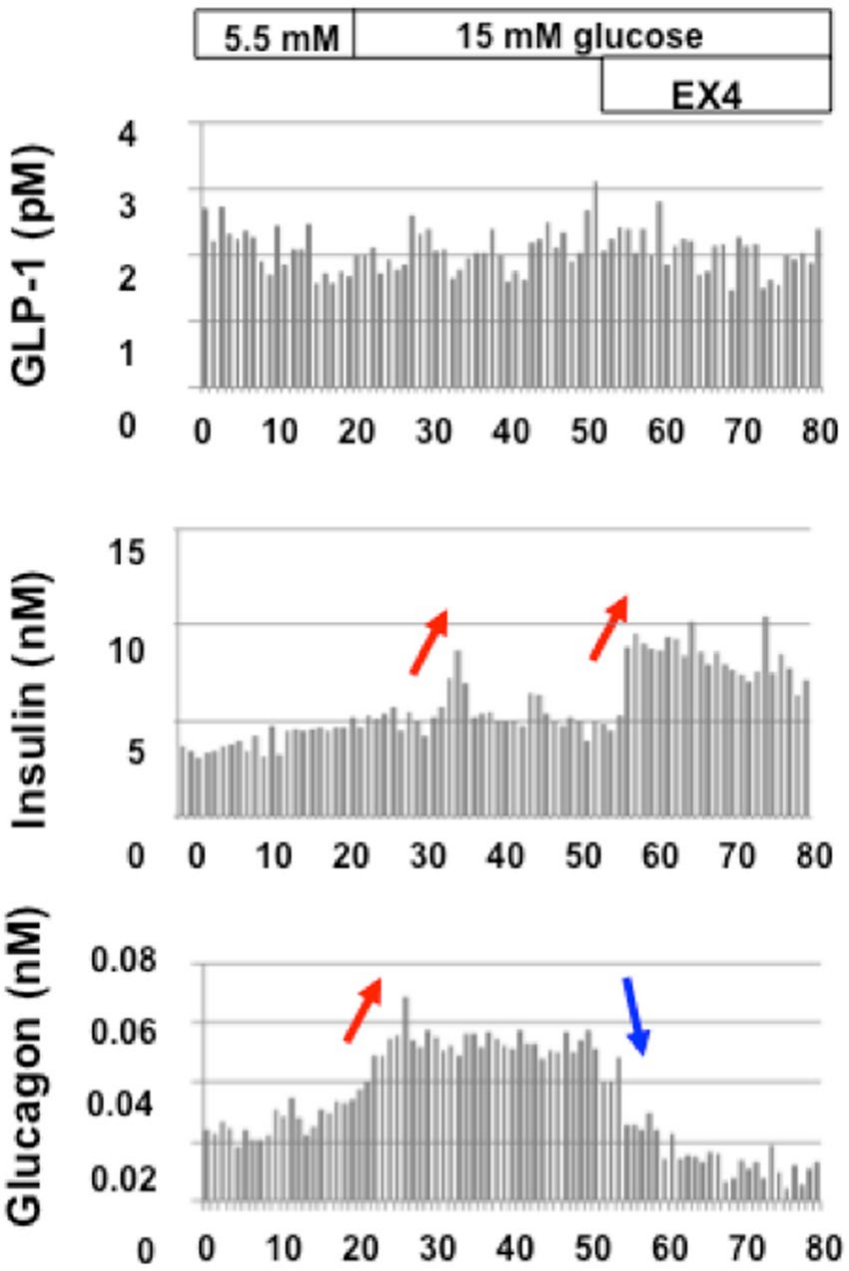
Isolated pancreatic perfusion profile of diabetic rats stimulated high glucose and EX4. 8-week-old GK rat (BW 272 g, BS 368 mg/dl). 1–20 min, 5.5 mM glucose; 21–60 min, 15 mM glucose; 61–80 min, 15 mM glucose and 10 nM EX4. Red arrows indicate elevation of GLP-1, insulin or glucagon secretion. Blue arrows indicate suppression of glucagon secretion.

Furthermore, as shown in Fig. 8, high glucose levels accelerated glucagon secretion, which could explain the paradoxical glucagon secretion under diabetic conditions^15–17^. Although insulin secretion was also enhanced, it was not sufficient to suppress the glucagon secretion. However, additional EX4 treatment further enhanced insulin secretion, which finally led to suppression of the glucagon secretion.

These data further support the idea that GLP-1 receptor is present in alpha cells and suggest that GLP-1 stimulates glucagon secretion in diabetic alpha cells. Glucagon secretion was suppressed by simultaneously secreted insulin in paracrine fashion.

## Discussion

GLP-1R is classified in family B of 7 transmembrane (7TM) GPCRs. It has two types of signaling pathway, classical and endosomal^34–37^. In classical (brief) signaling, binding of agonist ligand (GLP-1) to GLP-1R initiates signaling through the recruitment of heterotrimeric G proteins (αβγ). GDP-CTP exchange activates the Gα subunit which activates adenylate cyclase (AC) to generate a second messenger, cAMP. This classical signaling pathway is restricted to cell membrane, and sustained signaling is mediated by not only cAMP but also calcium and inositol phosphate. In endosomal (long) signaling, like many GPCRs, GLP-1R is then phosphorylated by GPCR kinae, leading to recruitment of and engagement with beta-arrestin. Beta-arrestin binding initiates receptor internalization into clathrin-coated pits by interaction with endocytic machinery. GLP1/GLP-1R signaling continues upon entry into early endosomes. Endosomal signaling induces prolonged cAMP responses for GLP-1/GLP-1R complexes to activate AC. This signaling is mediated by only cAMP.

Endosomes fall into three different categories: early endosomes (markers: EEA-1^38^, Rab5^46^), late endosomes (marker: Rab7^43,44^) and recycling endosomes (marker: Rab11^41,42^). Early endosomes mature into late endosomes before fusing with lysosomes. During maturation, some endosomes are trafficked in the cells to provide a signal as a signaling endosome^47,48^. Some endosomes become recycling endosomes for GLP-1R to transport back to the cell surface^41,42^.

In this study, we demonstrated that GLP-1 and GLP-1R were co-localized with endosome-related markers such as beta arrestin 2^39,40^ (Supple. Figure 3), EEA-1^40^ (Fig. 1), Rab11^41,42^ (Fig. 2 and Supple. Figure 4) and Rab7^43,44^ (Fig. 3) in diabetic islet cells. Beta arrestin 2^39,40^ appeared to recruit GLP-1/GLP-1R complexes into early endosomes, as observed in Supple. Figure 3, and EEA-1 appeared to fuse small endosomes to make large endosomes, as shown in Fig. 1c–f. As part of endosome maturation, Rab11 appeared to recycle GLP-1R to the plasma membrane, and large recycling endosomes contained the deformed structure, suggesting that GLP-1Rs were ready to recycle (Supple. Figure 4).

In large late endosomes (Fig. 3), GLP-1 expression was not observed, although cytoplasmic GLP-1 was stained in alpha cells (Fig. 3d white arrow), likely suggesting the formation of the hybrid organelle endolysosome to digest GLP-1 at first (Fig. 3) as a step in endosome maturation and a function of the endosome pathway. Only one small late endosome had faint GLP-1 (green arrow in Fig. 3) with red GLP-1R and blue Rab7 (percentage of GLP-1-positive endosomes, 6%). In early endosome GLP-1 was observed in 3 large and 4 small endosomes but was not detected in 1 large and 7 small early endosomes (GLP-1-positive, 59%, Fig. 1b, Supple. Figure 2). However, all recycling endosomes had GLP-1 (GLP-1-positive, 100%, Fig. 2b). These data suggest an endosome maturation process. Early endosomes without GLP-1 might maturate to late endosomes. Recycling endosomes might be returned to the cytoplasmic membrane at a relatively early stage.

These findings demonstrated the biologically important function and maturation of endosomes, indicating specific steps to isolate the GLP-1/GLP-1R complex into the endosomes in diabetic islet cells. In addition, these findings are consistent with the endosome system^34–38^, indicating that the endosomal co-localization study is reasonably useful. We could evaluate endosome maturation and function and establish novel staining of the GLP-1/GLP-1R complex. Using this method, we examined alpha cells to address the controversies about whether alpha cells have active GLP-1R. Finally, we demonstrated the presence of GLP-1R in alpha cells of diabetic rat, as shown in Fig. 4. We also showed the presence of AC^45^ in the alpha cells in diabetic rats (Fig. 5c,d). In addition, CLM specimens showed staining for AC, GLP-1 and Rab5 in normal and STZ-treated diabetic rats (Fig. 6). Because Rab5 is an early endosome marker^46^, AC and GLP-1 are incorporated into early endosomes. Figure 5 shows that AC, GLP-1 and GLP-1R were co-localized in islet cells. It is impossible to perform 4-colour staining at the same time by this method, so we examined two sets of staining: (AC, GLP-1 and GLP-1R) and (AC, GLP-1 and Rab5). Taken together, the results shown in Fig. 5 (AC, GLP-1 and GLP-1R) and Fig. 6 (AC, GLP1 and Rab5) indicate that AC was incorporated into early endosomes with the GLP-1/GLP-1R complex, suggesting that these endosomes work as signaling endosomes.

In addition, we confirmed that EX4, a GLP-1R agonist, markedly stimulated glucagon secretion during pancreas perfusion (Fig. 7c,d), suggesting the presence of active GLP-1R in the alpha cells of diabetic rats, although such glucagon secretion was immediately suppressed by GLP-1-stimulated insulin secretion from beta cells through an intra-islet paracrine mechanism, as reported previously^18^. There was some variation in the basal glucagon secretory capacity and/or maximal glucagon secretion amount, which presumably depended on the animals’ conditions. However, the glucagon secretion peaks were much higher than the basal glucagon secretion in all evaluated animals. These results strongly suggest that GLP-1 secreted from pancreatic alpha cells and/or intestinal L-cells bound GLP-1R on alpha cell membranes and that GLP-1/GLP-1R complexes were transported to the signaling endosomes. These findings clearly support the idea that GLP-1R is present and functions in the endosomes of glucagon-producing alpha cells in the diabetic condition, as demonstrated above.

It has been reported that GPCRs such as GLP-1R are difficult to detect immunologically using conventional immunocytochemistry and western blotting^30–33^ because GPCRs are 7TM receptors with low numbers *in vivo*, their epitopes are membrane-dependent small discontinuous segments, and they are heavily glycosylated molecules with highly conserved transmembrane domains. To solve this issue, in this study, we devised an endosomal co-localization study to detect the presence of active GLP-1R in pancreatic islet cells. Co-localization of ligand and GPCR pairs in endosomes indicates the binding of the ligand to its receptor. This situation seems plausible because we utilized a biologically specific process through which GLP-1R is recruited into the endosome and exists during the endosome maturation process.

Methodologically, we preferred cryostat sectioning rather than paraffin sectioning and did not use detergents or an antigen retrieval reagent to maintain the endosome structure. We used a GLP-1R antibody (Santa Cruz Biotechnology, Santa Cruz, USA. cat # sc-34637) that binds to the extracellular epitope to block GLP-1R activity^49^. GLP-1R and its supporter plasma membrane were incorporated into endosomes to maintain the extracellular epitope of GLP-1R. In addition, endosomal GLP-1/GLP-1Rs were free from dipeptidyl peptidase-4 or other proteases until late endosomes fused to lysosomes. Indeed, it was much easier to stain endosomal GLP-1/GLP-1R than the plasma membrane-bound GLP-1/GLP-1R in the tissue section. The cryostat pancreas section on the slide was fixed with 4% para-aldehyde at 4 °C for 15 min. This is a mild fixation prior to immune reaction.

Since we used no detergent or antigen retrieval reagent in this endosomal co-localization study to avoid destroying the structure of endosomes, in this system, it is very difficult to detect the expression of GLP-1R, which should be present on beta cell membranes. The epitope of GLP-1R in endosomes is preserved and recognized even without any detergent or antigen retrieval reagent. Therefore, the method in this study specifically detects GLP-1R in endosomes. In addition, we assume that cell adhesion to surrounding cells in our experimental sample might have impaired the detection of GLP-1R on the beta cell membranes. Indeed, as shown in Supple. Figure 5, GLP-1R was clearly detected in cell membranes in MIN6 cells that had no cell adhesion to surrounding cells. It was also reported that GLP-1R was observed on the cell membrane in a single cell of cultured cell line^46–48^.

On the contrary to the diabetic islets, endosomes with GLP-1 and GLP-1R complex were scarce in normal control islets (Figs 1a, 2a, 3a, 5a and 6a), nevertheless EX4 could stimulate insulin secretion (Fig. 7a). This discrepancy between immunohistochemical and physiological findings is not clearly assessed, but we speculate the following mechanisms.

1. Endocytosis is less activated in the normal islets compared to diabetic conditions. Up-regulation of endosomal GLP-1/GLP-1R complex in diabetic rats may be involved in an emergency pathologic process in which activated GLP-1/GLP-1R axis is required for their survival. While GPCR signaling is essential even in the normal conditiuon, continuos or over-stimulation may not be necessary. Accordingly healthy living cells have tightly regulated desensitization mechanism^50^. Therefore, it is likely that in normal islets, GLP-1 signal transduction is completed with GLP-1R on the cytoplasmic membrane (classical signal pathway)^34–37^. These findings may provide an important concept to understand a significance of GPCR research in diabetes condition.
2. In addition, we used antibody to react with epitope of extracellular GLP-1R domain (specific antibody to block GLP-1R activity)^49^. This method does not visualize GLP-1R on the cell membrane because the cell adhere each other to form tissue structure. GLP-1R was observed on the cell membrane of MIN6 cells because those do not adhere to other cells (Supple. Figure 5). It was also reported that GLP-1R was observed on the cell membrane in a single cell of cultured cell line^46–48^.

In the traditional immunohistochemistry, detergent and/or antigen retrieval agent solve cell membrane as well as endosome membrane to release GLP-1R, making it easy to react with antibody. However, the traditional method visualizes neither GLP-1/GLP-1R complex in the endosome nor GLP-1R on the cell membrane, thereby this method was not applied in this study.

Here, we hypothesize that the endosome system develops to potentiate GLP-1 signal transduction (endosomal signaling) in the diabetic state, while in physiologically normal condition, GLP-1 signal transduction occurs only on the cell membrane (classical signaling). Our method could provide an evaluation tool for endosomal signaling pathway recently established.

It is well known that under diabetic conditions, alpha cells paradoxically secrete glucagon, which leads to further aggravation of diabetes. Therefore, it is important to manage such undesirable glucagon secretion for the treatment of diabetes. It remains unclear, however, why alpha cells paradoxically secrete glucagon under diabetic conditions. In this study, we immunohistochemically found that GLP-1R was induced in alpha cells in diabetic rats. Furthermore, in the pancreas perfusion study (Fig. 7), we discovered that EX4 stimulated glucagon secretion, and simultaneously, the secreted insulin suppressed glucagon immediately (paracrine action) (Figs 7c,d and 8). In addition, as shown in Fig. 8, elevation of the glucose concentration from 5.5 mM to 15 mM induced glucagon secretion, which seemed to be involved in the paradoxical glucagon secretion under diabetic conditions. This glucagon secretion was suppressed by additional EX4 treatment. It is likely that the simultaneously secreted insulin directly suppressed glucagon secretion. Therefore, we assume that the induction of GLP-1R could explain why alpha cells paradoxically secrete glucagon under diabetic conditions. In other words, we think that GLP-1 binds to GLP-1R, which is induced in alpha cells in the diabetic state and paradoxically facilitates glucagon secretion from alpha cells.

There seems to be some difference in the underlying mechanism for hyperglycaemia between rodent and human type 2 diabetes. For example, it is known that hypercorticosteronaemia contributes to the diabetic phenotype in GK rats and should be considered a potential confounder in rodent models of type 2 diabetes^51^. Therefore, we think that the findings in this study are not necessarily true in humans and that further evaluation using human samples would be necessary to demonstrate this point. However, as we showed in this study, the GLP-1/GLP-1R complex was detected in the endosomes of pancreatic alpha cells in STZ-induced diabetic rats and GK rats. Therefore, it may be possible that the present observations are a relatively common phenomenon in hyperglycaemic conditions.

In summary, we presented a novel method to demonstrate the GLP-1/GLP-1R axis using endosomal co-localization staining with GLP-1 and GLP-1R. This method is especially useful to morphologically evaluate the endosomal signaling of GPCRs. Normal control rats have classical signaling for signal transduction of GLP-1 in beta cells. When the rats developed diabetes by STZ treatment, they express endosomal signaling pathway. GLP-1R expression was observed in alpha cells under diabetic conditions. We assume that the pancreatic GLP-1/GLP-1R complex functions through an intra-islet autocrine/paracrine mechanism. In addition, with pancreatic perfusion, a GLP-1R agonist facilitated glucagon secretion from alpha cells via GLP-1R, also supporting the idea that active GLP-1R is present in alpha cells under diabetic conditions and such phenomena are likely involved in the paradoxical hyperglucagonaemia in type 2 diabetes.

## Methods

### Animals

All male rats were purchased from CLEA, Tokyo, Japan, and fasted for 18 h before the experiments. Non-diabetic F344/Jcl and Wistar rats at the age of 10 and 25 weeks were used as the controls. To generate diabetic model rats, streptozotocin (STZ) was intraperitoneally injected twice (70 mg/kg weight/day) into 10–12-week-old F344/Jcl and Wistar rats, and 1 week later, the experiment was performed; other diabetic rats, 8- and 25-week-old GK rats, were also used. This study was approved by the Animal Use Committee of Kawasaki Medical School (no. 11–063) and was conducted in compliance with the Animal Use Guidelines of the Kawasaki Medical School.

### Immunocytochemistry

The rat pancreas was removed from the abovementioned treated control and diabetic model rats under sevoflurane general anaesthesia. The pancreas was then immediately mounted with OCT compound, frozen in liquid nitrogen and stored at −80 °C. A cryostat section (8 µm in thickness) was obtained to make glass slide samples. The specimen was fixed with 4% paraformaldehyde at 4 °C for 15 min and treated with 10 mmol/l glycine for 15 min. Blocking was performed with 2% donkey serum in PBS at 37 °C for 15 min, and the sample was then rinsed twice with 0.2% bovine serum albumin (BSA) in PBS. The primary and secondary antibodies are listed in Supple. Table 1. Primary staining was performed for 40 min at 37 °C by using a mixture of three primary antibodies diluted with PBS containing 2% donkey serum as indicated in Supple. Table 1. Samples were washed with 0.2% BSA in PBS for 5 min five times. Secondary antibody staining was then performed at 37 °C for 40 min by using three secondary antibodies selected for the primary antibodies. After the samples were washed 5 times, the specimens were sealed with Vectashield mounting medium (Vector Laboratorie, Burlingams, USA). A fluorescence image was taken with an LSM700 confocal laser microscope (Carl Zeiss Microscopy GmbH, Jena, Germany) using 405 nm, 488 nm, and 555 nm lasers for excitation and using BP400-470, BP490-555 and LP560 for emission. The image was processed using ZEN lite 2012 software (Carl Zeiss Microscopy GmbH, Jena, Germany) and Adobe Photoshop CS3. Simultaneously, the image was captured by a differential interference contrast (DIC) microscope (Carl Zeiss Microscopy GmbH, Jena, Germany) to confirm the endosome structure.

We evaluated how often GLP-1 was positive in the endosomes using original images and Photoshop cs3. In the magnified image in large display, we counted GLP-1 using split-merge function by channel soft of Photoshop. In this calculation, we hypothesized that 1 large endosome contained 4 small endosomes.

### Pancreatic perfusion study

A surgical operation was performed to serially tie and cut vessels from the rectum to the stomach to remove the stomach and intestine under sevoflurane general anaesthesia, and catheters were inserted into the abdominal aorta and the portal vein (Supple. Figs 9 and 10) ^6,52^. The pancreas was perfused with Krebs-Ringer-bicarbonate-HEPES with 5.5 mmol/l glucose, 3.0% dextran, 0.25% BSA, 40 nmol/l vildagliptin and 50 µg/ml aprotinin continuously gassed with a 95% O_2_ and 5% CO_2_ mixture at 37 °C through the celiac and superior mesenteric artery via a catheter inserted into the abdominal aorta. After the pancreas perfusion system was established, the rats were killed by cutting the thoracic aorta under deep anaesthesia. The perfusion study was started after pre-perfusion for 30 min. A peptide hormone stimulator, 10 nmol/l EX4 and 2 mmol/l 3-isobutyl-1-methylxanthine (IBMX, phosphodiesterase inhibitor) were added to the perfusate, as indicated in the figures. The perfusate from the portal vein was collected sequentially in 1 min intervals and stored at −80 °C until the measurements of GLP-1, insulin and glucagon were performed. The ELISA kits were GLP-1 (Active) ELISA (Merck Millipore, Darmstadt, Germany, cat # FGLP-35K), Glucagon ELISA kit (Yanaihara, Shizuoka, Japan, cat # YK090) and Insulin ELISA kit (Morinaga, Yokohama, Japan, cat # MS303).

## Electronic supplementary material

Supplementary Dataset
