# Supplementary figures and images for: Pancreatic alpha cells in diabetic rats express active GLP-1 receptor: Endosomal co-localization of GLP-1/GLP-1R complex functioning through intra-islet paracrine mechanism
Source: Sci Rep. 2018 Feb 27;8:3725. doi: 10.1038/s41598-018-21751-w (PMC5829082; doi:10.1038/s41598-018-21751-w)

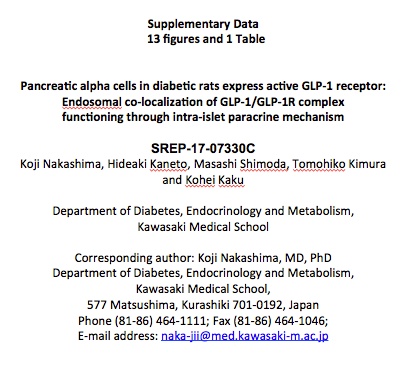


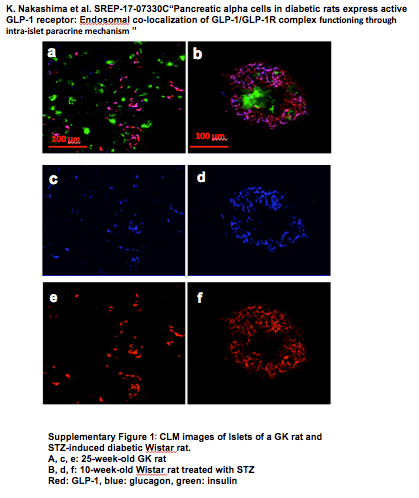


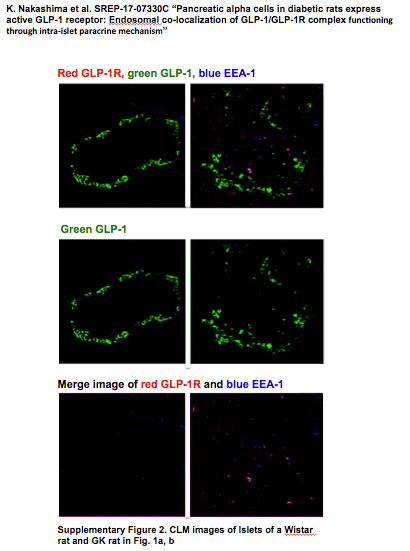


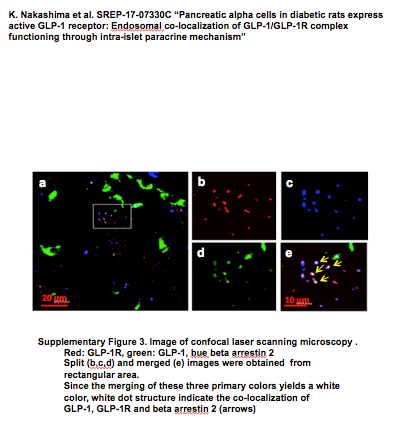


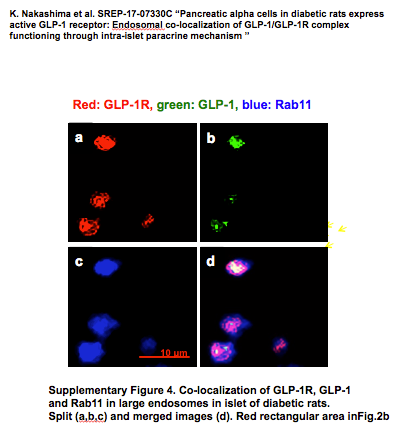


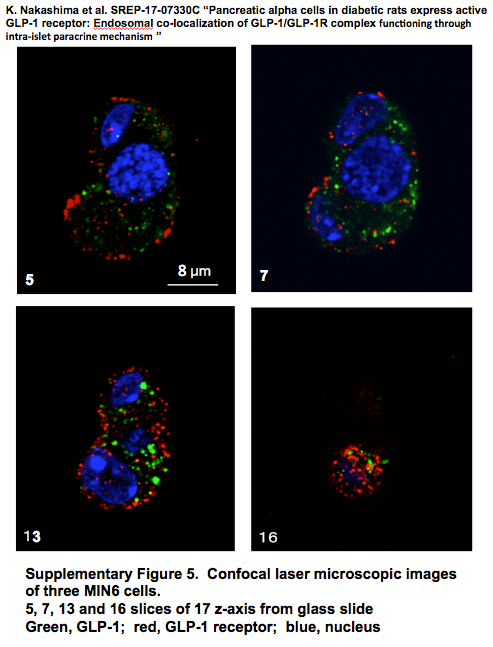


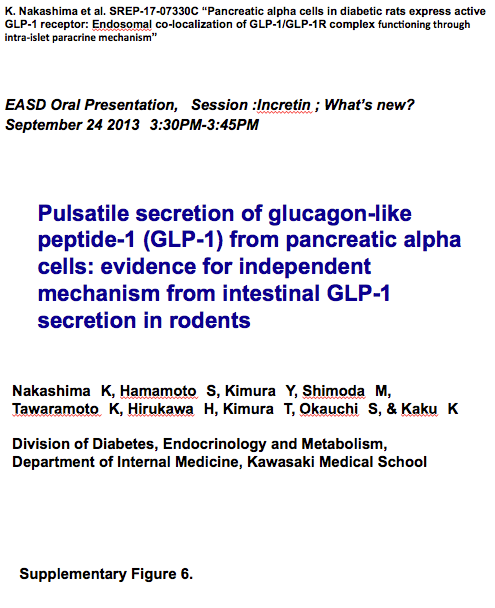


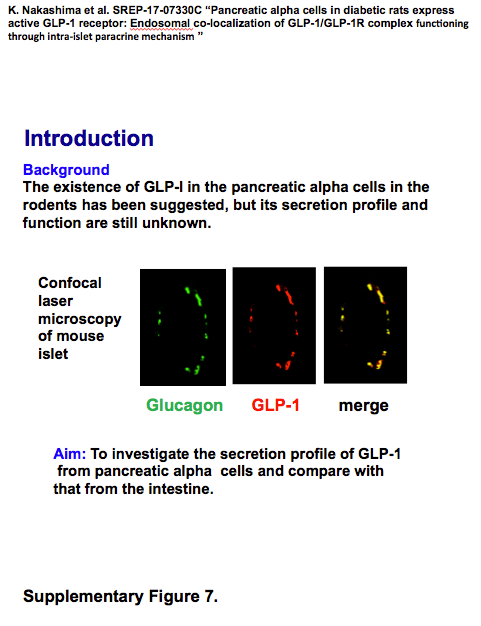


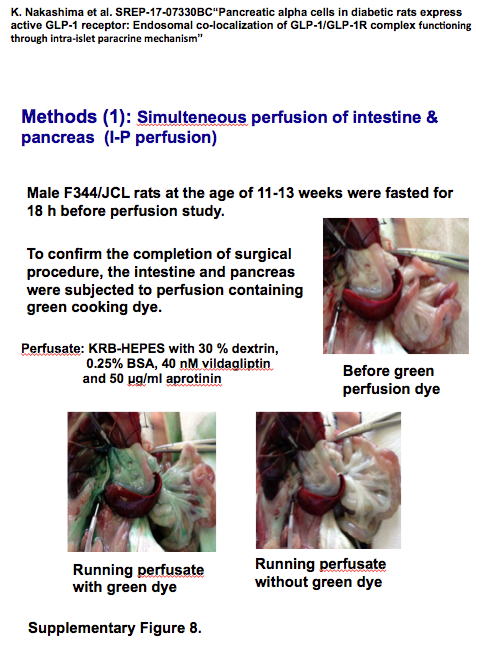


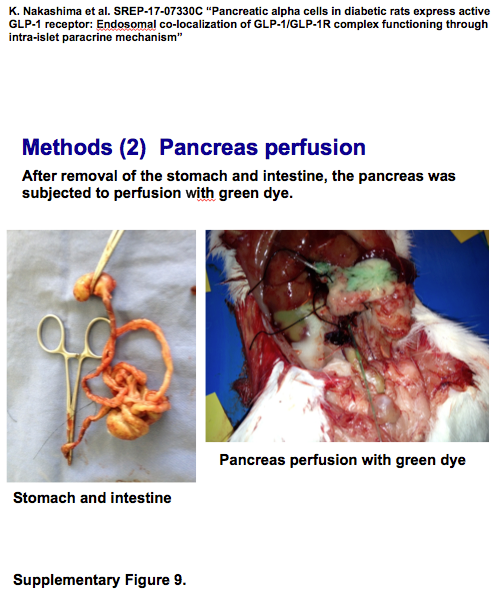


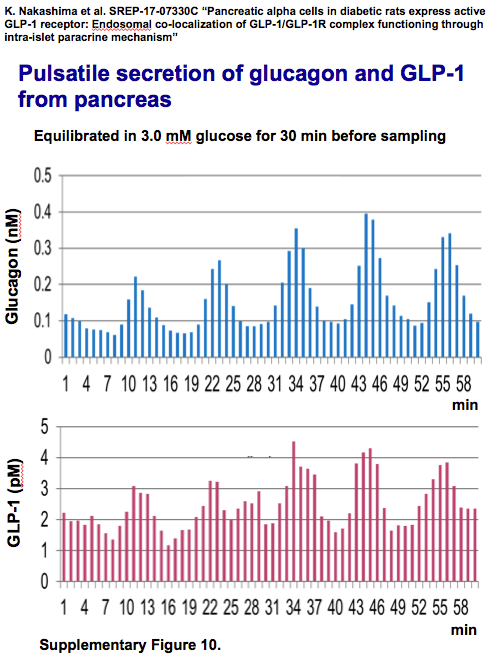


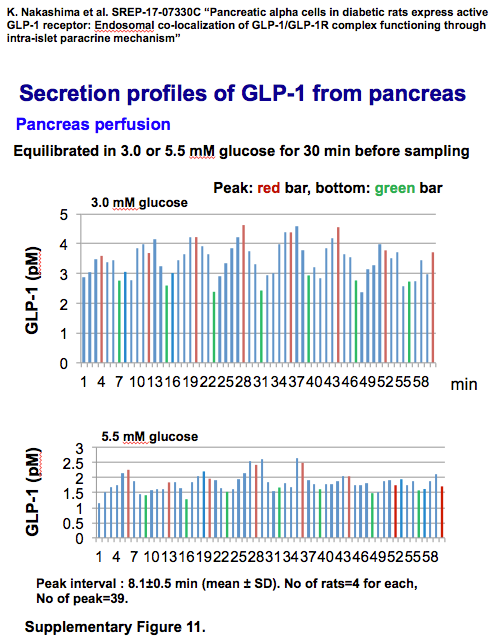


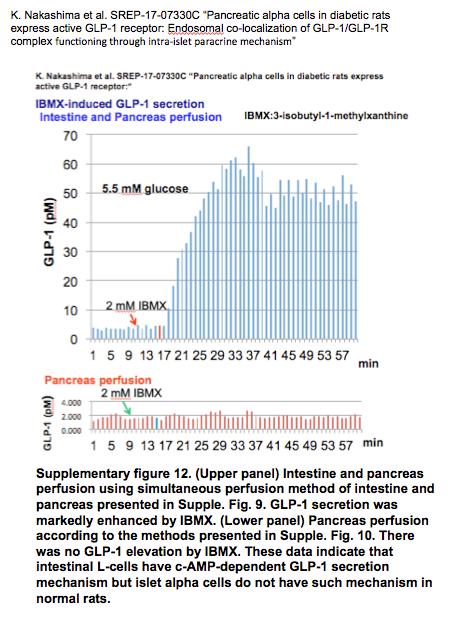


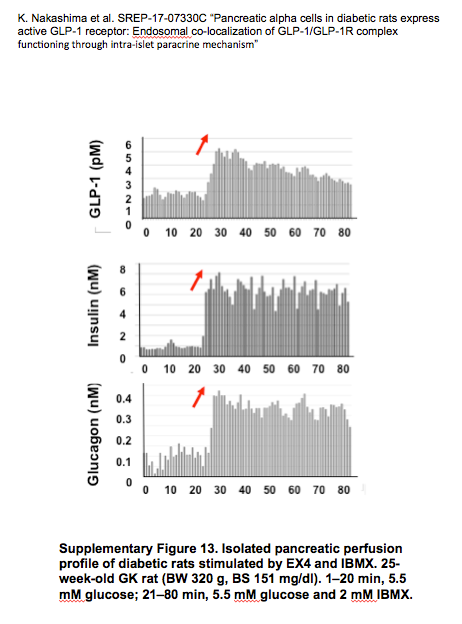


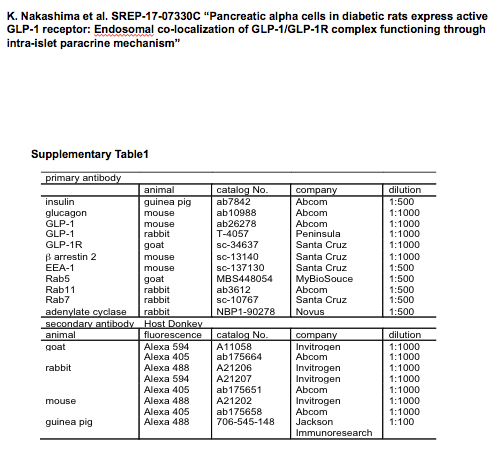

Supplement: Supplementary file 1 — Supplementary Dataset [file 41598_2018_21751_MOESM1_ESM.doc]
